# Interstitial 287 kb deletion of 4p16.3 including *FGFRL1* gene associated with language impairment and overgrowth

**DOI:** 10.1186/s13039-014-0087-2

**Published:** 2014-12-09

**Authors:** Eunice Matoso, Fabiana Ramos, José Ferrão, Luís M Pires, Alexandra Mascarenhas, Joana B Melo, Isabel M Carreira

**Affiliations:** Cytogenetics and Genomics Laboratory, Faculdade de Medicina da Universidade de Coimbra, Pólo Ciências da Saúde, 3000-548 Coimbra, Portugal; Medical Genetics Unit, Hospital Pediátrico, Centro Hospitalar e Universitário de Coimbra, Coimbra, Portugal; CIMAGO – Centro de Investigação em Meio Ambiente, Genética e Oncobiologia, Faculdade de Medicina da Universidade de Coimbra, Coimbra, Portugal

**Keywords:** 4p16.3 deletion, Developmental delay, *FGFRL1* gene, Language impairment, Cartilage formation

## Abstract

We report a male patient with developmental delay carrying an interstitial 4p16.3 deletion of 287 kb, disclosed by oligo array-CGH and inherited from his father with a similar but milder phenotype. This deletion is distal to the Wolf-Hirschhorn syndrome critical regions, but includes the *FGFRL1* gene proposed to be a plausible candidate for part of the craniofacial characteristics of Wolf-Hirschhorn syndrome patients. However, the proband lacks the typical facial appearance of the syndrome, but exhibits overgrowth, dysfunction of temporomandibular articulation and a bicuspid aortic valve. Given the pattern of expression of the *fibroblast growth factor receptor-like* 1 and its involvement in bone and cartilage formation as well as in heart valve morphogenesis, we discuss the impact of its haploinsufficiency in the phenotype.

## Background

Wolf-Hirschhorn syndrome (WHS; OMIM#194190) is a contiguous gene syndrome caused by deletion of the short arm of chromosome 4. There is a considerable variation in the phenotypic spectrum and a correlation between the size of the deletion and severity of the phenotype has been recognized [1]. Two Wolf-Hirschhorn critical regions, WHCR1 and WHCR2, have been identified within 4p16.3 [2,3]. Beyond this region, the loss of additional critical genes appears to be responsible for variable present features, the identification of patients with distal 4p deletions could help to identify for pathogenic genes that may be involved in components of the WHS phenotype.

Here we report a patient with a mild phenotype carrying an interstitial 287 kb deletion at 4p16.3, distal to the already described WHS critical regions, and encompassing *FGFRL1* gene proposed to be involved in the craniofacial characteristics. We discuss the involvement of this gene in the features displayed by the patient and compare with other patients reported with overlapping imbalances.

## Case presentation

### Clinical report

**Figure 1:**
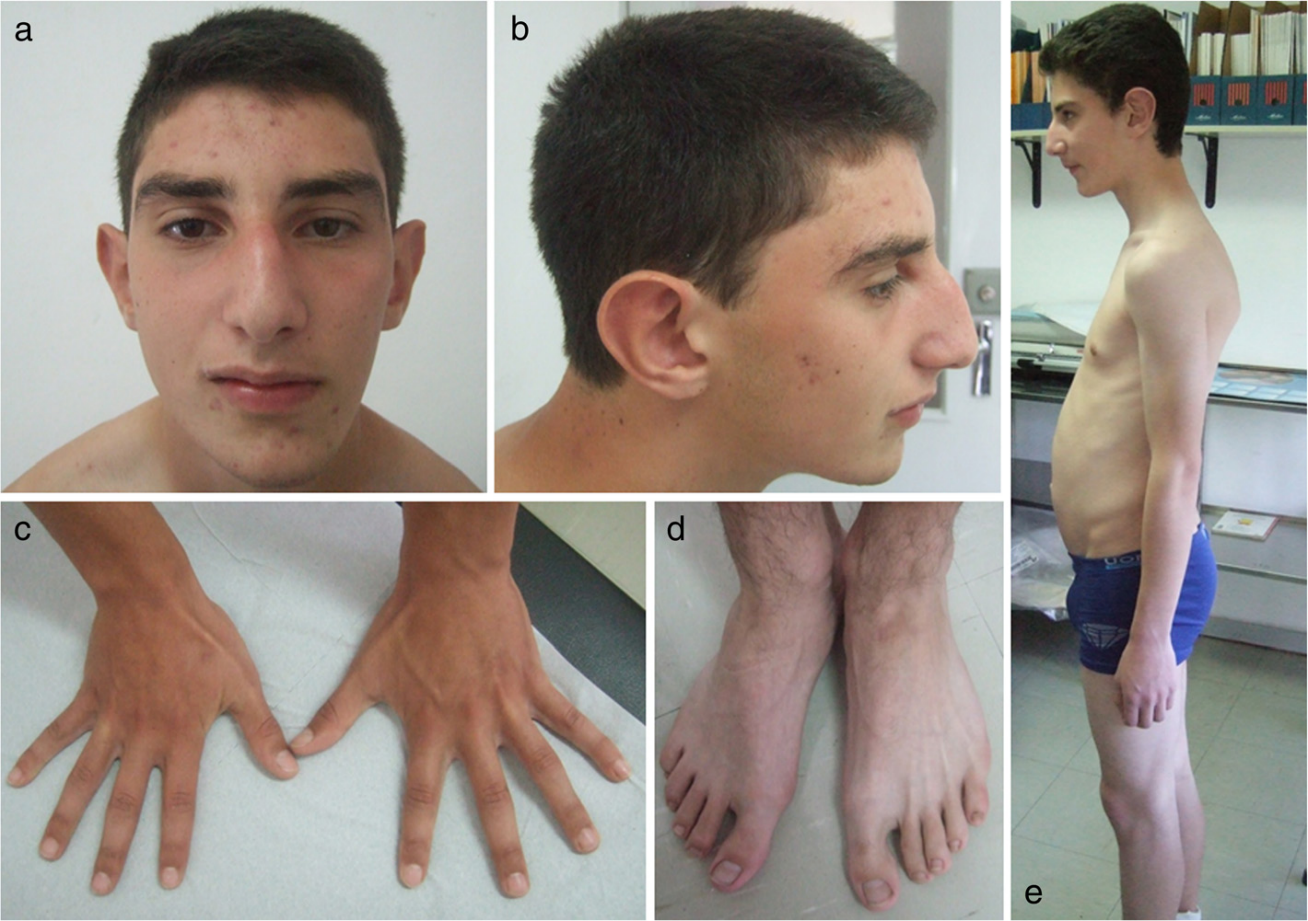
**Phenotypic characteristics of the patient at 14 years of age. (a)** Asymmetric long face, with retrognathia and short philtrum, **(b)** long and prominent nose, large or prominent ears, **(c)** hands with long fingers, **(d)** bilateral sandal gap of the first toes with large halluces, **(e)** postural kyphosis with sloping shoulders.

The 11-years-old male proband is the first child of unrelated parents. He was born at 38 weeks of gestation after uneventful pregnancy. His weight was 3900 g (90th percentile), length of 51 cm (50th percentile), and occipitofrontal circumference (OFC) of 36 cm (75th percentile). Apgar scores were 9 and 10 at 1 and 5 minutes, respectively. He started to talk and walk independently at 2 years of age. It was noted to have difficulties in language skills, so he has been having language therapy since the age of 3. At 6 years he started to have pedo-psychiatric support. At the age of 11 years he was referred to genetics evaluation after diagnosis of bicuspid aortic valve in routine echocardiography. On physical examination, the patient showed overgrowth with a weight of 51,5 kg (95th percentile), height of 168 cm (>95th percentile), and OFC of 53,5 cm (50th-75th percentiles). Craniofacial findings included an asymmetric long face, with retrognathia, short philtrum (Figure 1a), high palate with misaligned teeth, dysfunction of temporomandibular articulation, long prominent nose and prominent ears (Figure 1b). The hands have long fingers (Figure 1c), the feet display bilateral sandal gap of the first toes with large halluces (Figure 1d). Because of the developmental delay and overgrowth, homocysteine levels were investigated but showed a normal result. Electroencephalogram was normal, but MRI of the brain revealed a slight diminish of the frontal lobe mainly on the right side. Although in orthopedic examination he didn’t reveal any remarkable alterations, he has a postural kyphosis with sloping shoulders (Figure 1e) and asymmetric position of scapulas. He has a mild global cognitive impairment, but a more pronounced dysfunction in expressive language and articulation of speech.

His father has similar but milder phenotypic features and had strong learning difficulties at school, also with limitations in language articulation. He exhibits a tall stature, the facial appearance displays a slight asymmetry with a long prominent nose, short philtrum and retrognathia. Neither the patient nor his father have had seizures.

In the father’s family there is a history of remarkable delay in his sister, brothers and nephews with learning difficulties. Unfortunately, it was not possible to evaluate any other of the affected family members.

The proband had already normal investigation results of conventional cytogenetics analysis, fragile-X syndrome testing and subtelomeric screening.

## Methods

High-resolution comparative genomic hybridization (array-CGH) analysis was performed using Agilent SurePrint G3 Human Genome microarray 180 K (Agilent Technologies, Santa Clara, CA), an oligonucleotide microarray containing approximately 180,000 sixty-mer probes with a 17 kb average probe spacing, as described before [4]. In order to confirm the array-CGH result, MLPA (MRC Holland, Amsterdam, Netherlands) using the set of probes P373, was performed according to the manufacturers’ instructions. The study of the parents was done with the same MLPA probemix.

## Results

**Figure 2:**
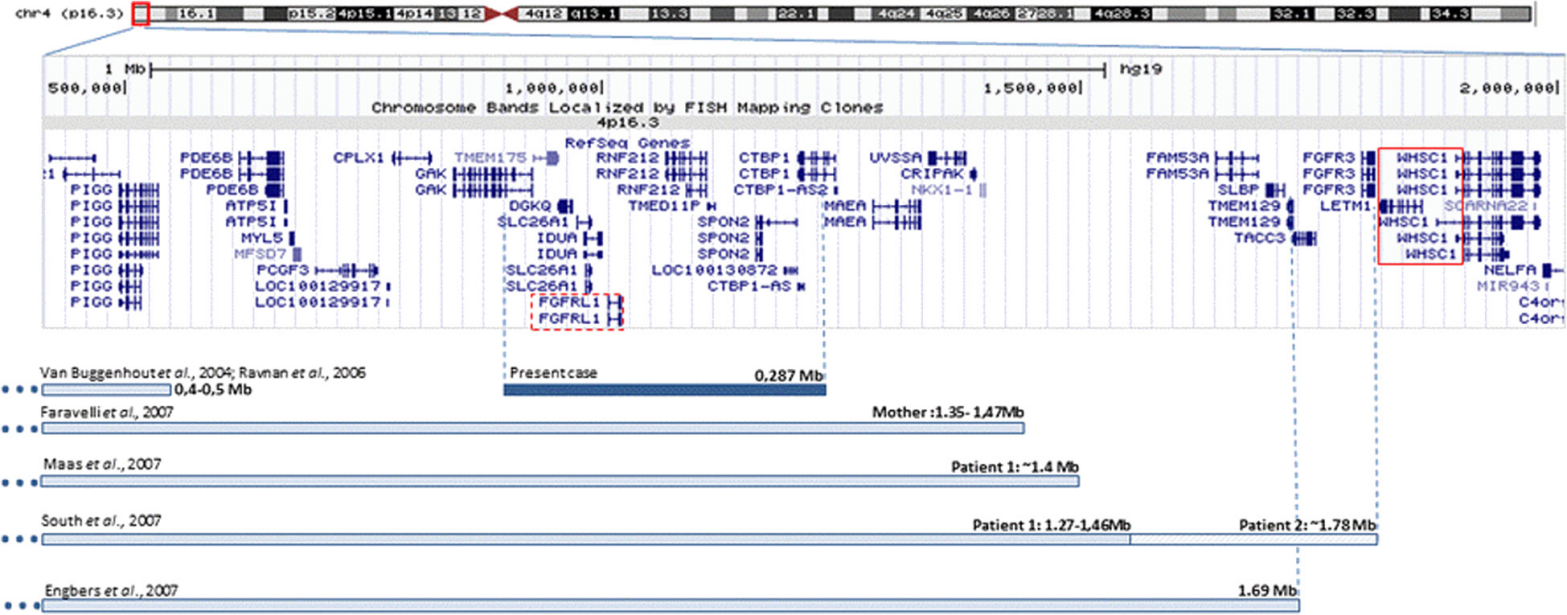
**Genomic region of 4p16.3 involved in the deletions of patients with distal imbalances to WHS critical regions (adapted from UCSC genome browser, GRCh37/hg19), displaying the RefSeq genes.** A comparison of the extension of the deletions with previously reported patients is also shown.

The oligo array-CGH showed an interstitial deletion at 4p16.3 spanning about 287 kb of genomic DNA from position 927,780 bp (clone A_16_P16565674) to 1,214,915 bp (clone A_16_P00907084) flanked by probe A_16_P00906727 (920,051 bp) and probe A_18_P23419088 (1,228,462 bp) according to UCSC Genome Browser (hg19; GRChBuild 37.1, February 2009) (Figure 2). The deleted segment contains 11 RefSeq genes, 7 genes are described in the OMIM database: *DGKQ* (#601207) a diacylglycerol kinase, *SLC26A1* (# 610130) a sulfate anion transporter 1, *IDUA* (# 252800) a alpha-L-iduronidase (associated to a lysosomal storage disease by homozygous or compound heterozygous mutation), *FGFRL1* (#605830) the fibroblast growth factor receptor-like 1, *RNF212* (#612041) a ring finger protein that plays a role in meiotic recombination, *SPON2* (#605918) the spondin 2, *CTBP1* (#602618) the C-terminal-binding protein 1.

MLPA confirmed the array result with two probes for genes *SPON2* and *FGFRL1.* Parental testing was performed with MLPA, showing the father to be carrier of the same imbalance.

## Discussion

We report a patient with an interstitial deletion on 4p16.3, inherited from the father, this deletion is distal to de WHS critical regions. However, this imbalance encompasses the *fibroblast growth factor receptor-like 1* (*FGFRL1)* gene proposed to be an important candidate contributing to the craniofacial phenotype [5,6]. The patient described lacks the typical facial features of the WHS, only exhibiting prominent nose and short philtrum. This gene has also been associated with intrauterine growth retardation and postnatal short stature in WHS patients [7], opposite to the finding in our patient that displays overgrowth since the neonatal period. Delayed in speech is a frequent problem in patients with 4p16.3 deletions [8], even among patients with non overlapping imbalances. Probably more than one gene contributes to the language impairment, that in the proband and his father could be associated with the dysfunction of the temporomandibular articulation.

FGFRL1 inhibits cell proliferation, but promotes cell differentiation and induces cell adhesion (involved in cell-cell fusion), it is primarily expressed in cartilaginous tissues and in bone primordia (like the maxillae, the mandibles, the ribs and the nose), the muscles of the tongue and the diaphragm express *FGRFL1* at a relatively high level, but the heart, aorta, skeletal muscle, brain, lung, liver, kidney and gut it is expressed at a moderate level [9]. Taking into account its function and pattern of expression, the haploinsufficiency of *FGFRL1* could be associated with the craniofacial phenotype of proband including the dysfunction of temporomandibular articulation and the overgrowth could be a consequence of the partial lack of the negative effect of FGFRL1 in cell proliferation particularly in bone tissues. However, in the research work of Catela *et al. *[6] with an *Fgfrl1*^*−/−*^ null mice, an animal model to dissect the aetiology of WHS, as far as we know overgrowth has never been detected, only short stature is described in mutants that develop to term, but most homozygous embryos suffer prenatal death. Since the heterozygous mutant *Fgfrl1*^*+/−*^ did not display a discernible phenotype [6], these contradictory results are difficult to interpret. Probably in humans the haploinsufficiency of other neighbouring genes plays a critical role in the physiological impact of *FGFRL1* heterozygous deletion. The presence of cardiac defects is observed in some patients with WHS [7], but a patient described by Okamoto *et al.* [10], with a small deletion encompassing *WHSC1* but not including the *FGFRL1*, didn’t exhibit cardiac malformation. On the other hand, in the report of South *et al*. [11] the echocardiogram of both patients didn’t reveal any alteration although the deletions involved include the *FGFRL1* gene. In the patient described the presence of a bicuspid aortic valve could be a coincidental finding or maybe suggests the contribution of FGFRL1 in heart valvulogenesis. This observation is supported by the experimental results with the *Fgfrl1*^*−/−*^ null mice, suggesting a role for Fgfrl1 in valvulogenesis and ventricular septation [6].

In the work reported by Hammond *et al.* [12] a correlation between the genes involved in different deletions within 4p16.3 and the craniofacial phenotype is done. One of the patients was reported by Faravelli *et al.* [13], mother of the propositus, she carries a terminal deletion distal to both critical regions of WHS, but including *FGFRL1*, her facial features included hypertelorism, high nasal bridge, down-slanting of palpebral fissures and large protruding eyes, but our patient with a smaller deletion also harbouring *FGFRL1* doesn’t display neither of these characteristics. South *et al*. [11] described two patients with 4p terminal deletions distal to WHS critical regions but including *FGFRL1* and a portion of *LETM1* in one of them (Figure 2), they didn’t exhibit the typical facial features of the syndrome, but have prominent forehead, mild telecanthus and normal philtral length, in both it was observed growth delay and mild developmental delay. On the other hand the patient reported by Engbers *et al*. [5] with a 1.6 Mb deletion have typical facial signs of the WHS, this deletion includes *FGFRL1* but also other proximal genes that could be involved in the displayed phenotype (Figure 2). Our report supports the hypothesis that multiple genes are involved in the orchestration, not only of the facial phenotypic features but in the other malformations and cognitive skills observed in WHS patients in a contiguous manner. As it was confirmed in the study of Maas *et al.* [7], of a cohort of patients with different size deletions and genotype-phenotype correlation, several clinical features of WHS have considerable variable expressivity or penetrance, so it is difficult to pinpoint the genes involved in the more rare aspects of the WHS phenotype. The identification of distal 4p deletions covering about 0.4-0.5 Mb in individuals without a pathogenic phenotype suggests that monosomy of this region is likely benign [14,15]. But deletions extending to the 1.4 Mb region appear to be pathogenic (Figure 2), although with a mild phenotype [7,11,13] and no typical facial appearance of WHS. Deletions extending to 1.6 Mb can however display some overlapping features to the phenotype of WHS [5], suggesting the presence of other genes contributing to the displayed features.

## Conclusions

The present report could help to unravel the phenotypic manifestations underlying the haploinsufficiency of the *FGFRL1* gene. Its involvement in the bone primordia formation and in heart morphogenesis has been described even in animal models [6]. However, the observation of a tall stature in the proband and his father is a specific finding of this report. A better characterization of patients with atypical and smaller interstitial deletions could contribute to the knowledge of the biological function of neighbouring genes out of the core critical regions of the WHS.

## Consent

Written informed consent was obtained for publication of this paper and any accompanying images. A copy of the written consent is available for review by the Editor-in-Chief of this journal.
